# Role of C-Jun N-Terminal Kinases on a Stressed Epithelium: Time for Testing Isoform Specificity

**DOI:** 10.3390/biology14060649

**Published:** 2025-06-03

**Authors:** Nitesh Shashikanth, Osama Alaidi, Lohitha Basa, Shreya Taank, RadhaKrishna Rao, Jayaraman Seetharaman

**Affiliations:** 1 Department of Physiology, University of Tennessee Health Science Center, Memphis, TN 38103, USA; nshashik@uthsc.edu (N.S.); lbasa@uthsc.edu (L.B.); shreyadta03@gmail.com (S.T.); rrao2@uthsc.edu (R.R.); 2 Department of Pharmaceutical Sciences, University of Tennessee Health Science Center, Memphis, TN 38163, USA; oalaidi@uthsc.edu; 3 Veteran Affairs Medical Center, Memphis, TN 38104, USA; 4 Department of Pharmacology, Addiction Science and Toxicology, University of Tennessee Health Science Center, Memphis, TN 38103, USA

**Keywords:** stressor, reactive oxygen species, c-Jun N-terminal Kinase (JNK), epithelium, inhibitors, kinase, structure–function relationship

## Abstract

Biological, physiological, and psychological stressors cause a “stress response” in our bodies. Stressors that are sensorily perceived (either acute or chronic) trigger hormonal responses from the sympathetic nervous system—the SAM and HPA axis—that effect intended organs to alert the individual. Other stressors have a direct effect on the target organ(s) of the body—e.g., physical injury and wounds, toxins, ionizing, and UV radiation. Both kinds of stressors change cell equilibrium, often leading to reactive oxygen species (ROS) accumulation and cellular damage. Among the signaling pathways involved in fighting these stressors, the c-Jun-N-terminal kinases (JNK) respond to diverse kinds of stressors. This review focuses on JNK1 and JNK2, both of which are ubiquitously present in all cell types, and attention is paid to gastrointestinal tract epithelial cells and their response—including tight junction disruption and cytoskeletal changes. We discuss the seemingly opposite roles of JNK1 and JNK2 in helping cells choose pro-survival and pro-apoptotic pathways. We examine the common features of the JNK protein structure and the possibilities of discovering JNK-isoform-specific inhibitors since, although JNK1 and JNK2 are involved in multiple diseases, including cancer, obesity, diabetes, musculoskeletal and liver disease, no cell-specific or isoform-specific inhibitors are available.

## Simple Summary

Stress, whether it is biological, psychological, or physical, manifests in the body as chemical changes. Perceived stressors affect neurotransmission that alter how organ systems react, leading to either a fight-or-flight response or development of chronic stress. Environmental exposure-based stressors, like toxins, pollutants, ultraviolet and ionizing radiation, low levels of oxygen like the deep sea, etc., can cause “oxidative stress” wherein the oxygen is not successfully converted to energy but accumulates as free radicals, like hydrogen peroxide and superoxide. This accumulation is often harmful to the affected cells, which mount a response by triggering many signaling pathways leading to either cell survival or cell death depending on the pathway activation. We discuss one such pathway, termed “c-Jun N-terminal Kinases” or JNKs. We focus this review on how JNKs play potentially distinct roles in different cell types to the same kind of stress that exacerbates disease. Targeting these kinase enzymes with inhibitors specific to their different isoforms could be a useful strategy in combating chronic stress-related diseases.

## 1. Introduction

**Figure 1:**
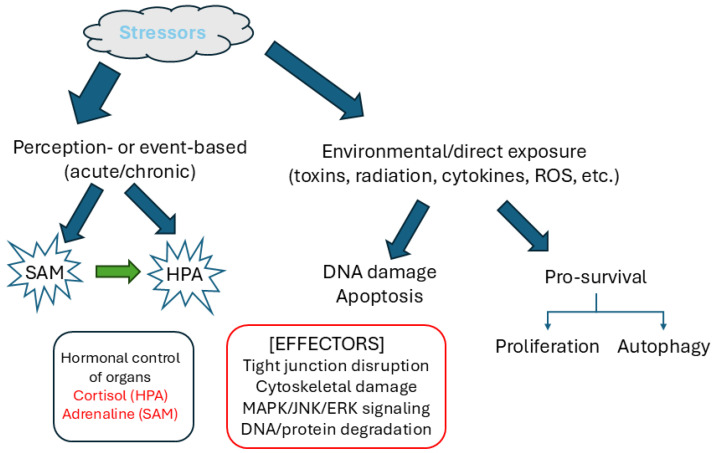
Stressors and stress response. Stressors are either perceived threats that triggers the nervous system to activate either the SAM or HPA axis, which releases hormones into the bloodstream, and it also controls organ function to enable coping with stressful circumstances or direct environmental exposures, like toxins, radiation, physical injuries, etc., which cause direct organ damage. The epithelial cells lining many of these organs are affected by ROS release due to stressor-associated damage, thereby causing epithelial barrier dysfunction by tight junction disassembly, actin cytoskeleton constrictions, and, finally, apoptosis, which is signaled by a variety of GTPases and kinases. The signaling can also tilt the cell fate toward autophagy or to activate other pro-survival or growth signals.

Stress is a physiological, biological, or psychological response to environmental events/stimuli known as stressors. Sensory perception of acute stress (fight-or-flight situations) triggers signaling through the sympathoadrenal medullary (SAM) axis of the sympathetic nervous system [1,2]. The hormones norepinephrine and epinephrine (noradrenaline and adrenaline) are released directly into the bloodstream, which induces rapid physio-psychological changes. In contrast, the hypothalamic–pituitary–adrenal (HPA) axis is involved in a short-to-long-term response in releasing cortisol, which regulates metabolic, psychological, and immunological functions [1,3]. In response to stress, the hypothalamus releases Corticotropin-releasing hormone (CRH), stimulating the pituitary to produce the Adrenocorticotropic hormone (ACTH), which prompts the adrenal cortex to release cortisol. This stress-induced hormonal release (SAM or HPA axis mediated [2,4]) translates to an effector response in the target organs (Figure 1). The type of effect depends on the biochemical properties of the cells that make up the tissues of these organs. Stress activators cause stress responses that are felt by nearly every major organ and organ system, including the cardiovascular, respiratory, musculoskeletal, immune, and gastrointestinal systems [1,2,3,4,5].

Many stressors cause an automatic response directly at the cellular level of the targeted/affected organs. For example, exposure of the skin to ultraviolet (UV) radiation triggers melanin production. Whether autonomous or hormone-induced, stress at the cellular level is broadly defined as the molecular changes that occur due to events that stress the organism [6]. Extrinsic stimuli are stressors like physical trauma that cause external and internal wounds that directly damage tissues. This includes UV, ionizing radiation, nutrient deprivation [7] or excess [8], infections, and exposure to poison/toxins. Intrinsic stimuli are from within an actively functioning cell that derails from homeostasis. This stems from the accumulation of unfolded proteins in the endoplasmic reticulum, which is also called ER stress. Stressors trigger an “intrinsic stress response” in cells that causes immediate downregulation of protein synthesis and upregulation of stress response-activated genes [9].

Various kinds of stress elicit different signaling pathway responses that direct cells toward seemingly opposite fates: either a pro-survival response or a pro-apoptotic response (Figure 1). Pro-survival responses attempt to recover cells from damage, such as, for example, the DNA damage repair pathways activated by UV radiation. Pro-apoptotic response directs activation of apoptosis pathways to eliminate damaged cells—depending on the kind of stressor and the cell type. Continuous exposure to the stressors impairs these responses. Pro-survival signals cascade into pro-oncogenic pathways while continuous cell damage impairs the function of the concerned organs. Studying stress-activated signaling mechanisms is thus crucial to reverse or prevent damage mediated by stressors.

This review will focus on c-Jun N-terminal Kinases (JNKs), formerly known as stress-activated protein kinases (SAPKs). JNKs are distinct from other mitogen-activated protein kinases (MAPKs) due to their activation by stress signals, such as cytokines, oxidative stress, and UV radiation, rather than by growth factors or mitogens [10].

The ability of cells to interpret and respond to stress is essential for maintaining tissue integrity and function. In these responses, JNKs serve as molecular switches that determine whether a cell adapts, repairs damage or undergoes apoptosis. By examining the diverse stressors that activate JNK signaling, their molecular mechanisms, and their broader physiological consequences, this review aims to uncover how JNKs act as both protectors and potential drivers of disease.

## 2. Cellular Damage Stressors

Cells are in a constant state of vigilance, responding to both external and internal threats that challenge their stability. Stressors, whether external, like radiation and oxidative damage, or internal, such as metabolic dysfunction and protein misfolding, trigger intricate molecular defense mechanisms. While many cellular responses are protective, prolonged (chronic) and/or severe stress can shift cellular outcomes toward dysfunction. At the core of these mechanisms are JNKs, which can integrate diverse types of stress signals.

i. **Reactive Oxygen Species (ROS)-mediated oxidative stress**: Endogenously, peroxides (O2•−) and superoxide radicals are byproducts of the mitochondrial electron transport chain (ETC) [11,12,13]. Approximately <3% of the electrons involved in oxygen reduction prematurely escape, forming superoxide, peroxide, or hydroxyl free radicals that diffuse across the mitochondrial membrane [14,15,16]. The presence of free radicals activates free-radical quenching enzymes like catalase, superoxide dismutase, and peroxidases that reduce the ROS burden and ROS-induced damage. However, excessive ROS accumulation due to exogenous stressors can overwhelm protective mechanisms. This leads to a response known as oxidative stress, which ultimately causes disruption of cell–cell integrity and localized and systemic inflammation [11,17,18,19,20,21]. In the gastrointestinal epithelium, it leads to tight junction disruption followed by endotoxemia, overexpression, and secretion of inflammatory cytokines, and it also transforms localized inflammation into a more systemic response [22,23,24]. ROS can be directly produced from the ETC or by various kinds of other extrinsic stressors that are mentioned below.
ii. **Osmotic stress**: Osmotic stress occurs due to steep concentration gradients across a barrier. Osmotic stress is associated with loss of intestinal barrier integrity and increased paracellular flux (flow of ions and macromolecules through the space between two adjacent cells). Human plasma osmolality is tightly maintained at 277–299 mOsm/kg [25]. Any potential fluctuations can affect cell volume and function. Exposure to a hypo-osmotic environment results in fluid flux into the cell, causing it to swell. Hyper-osmotic conditions can trigger cellular dehydration and shrinking. Both hypo- and hyper-osmotic states affect the colon. Hyperosmotic stress can specifically cause expressions of ROS and proinflammatory cytokines that trigger immune reactions [26,27,28]. Osmotic stress has been shown to disrupt tight junctions, remodel the actin cytoskeleton, and activate JNK signaling in intestinal epithelium in vitro with a Caco2 monolayer model [29].
iii. **Radiation exposure**: Both short- and long-wavelength radiation can induce cellular stress. Ionizing radiation, such as X-rays and γ-rays, generate ROS almost immediately upon tissue exposure, leading to widespread oxidative damage. ROS levels increase more than 10-fold upon ionizing radiation exposure [21,30,31]. UV radiation exposure, on the other hand, primarily stresses the cells by causing DNA damage that outpaces the regular levels of DNA repair that occurs during normal homeostasis [20,32]. This imbalance can lead to genomic instability, mutations, and cell death.
iv. **Hypoxia**: Lack of oxygenated blood and, therefore, oxygen supply to all organ systems causes severe stress on all vital organs. External stressors, like high altitude, deep-sea environments, or strenuous exercise, lead to inadequate amounts of oxygen consumption by tissues. Under low oxygen conditions, mitochondria become less efficient in electron transfer to water, leading to ROS formation and, consequently, oxidative stress. In response, cells activate hypoxia-inducible factor (HIF) proteins [33,34,35,36], which shift metabolism toward anaerobic energy production, promote angiogenesis, and modulate inflammation to enhance oxygen uptake and survival.
v. **Metabolic stress**: Metabolic stress arises when cells experience deficiencies or excesses in micro- or macro-nutrients. This disrupts energy balance and biochemical homeostasis. It can also occur due to the excess accumulation of nutrients, like glucose. In Type-2 diabetes, glucose accumulation leads to glucotoxicity and ROS production, leading to a buildup of cellular stress [13,37,38]. Diseases that affect organs involved in digestion, like pancreatitis, lead to a decrease in citrulline concentration, which causes severe metabolic stress. Interestingly, nutrient starvation has been explored as a therapeutic strategy in cancer as rapidly dividing tumor cells are particularly sensitive to L-glutamine deprivation, which can impair their growth and survival more than that of primary cells [7].
vi. **Heat Shock Response (HSR)**: The heat shock response is triggered by sudden temperature increases, which induce protein misfolding and ER stress [39]. To counteract this, cells upregulate heat shock proteins (HSPs). These function as molecular chaperons to stabilize misfolded proteins, prevent aggregation, and restore proper protein folding [40,41,42]. Without this protective response, heat-induced stress can cause protein dysfunction and ER overload.
vii. **Endoplasmic Reticulum (ER) stress**: ER stress occurs when the protein-folding capacity of the ER is overwhelmed by intrinsic genetic mutations, excessive protein synthesis, or disruptions in calcium homeostasis [43]. To mitigate this, cells activate the ER-associated degradation (ERAD) pathway [44], which clears misfolded proteins to restore normal function [45,46]. However, if ER stress is prolonged or unresolved, it can trigger apoptosis through the unfolded protein response. ER stress is implicated in various metabolic and genetic disorders, including Type-2 diabetes, neurodegenerative disease, and cancer. Furthermore, heat shock can exacerbate ER stress and upregulate HSPs to manage protein accumulation [47].

While the different “categories” of cellular stress mentioned above may seem isolated from each other, the signaling mechanisms that they trigger most often overlap. For many modes of stress (metabolic, radiation, osmotic, and hypoxia), ROS accumulation is the most common among the stressors, which ultimately leads to signaling responses.

## 3. Oxidative Stress and Barrier Function

The epithelial cells of the skins and tubular organs form a single sheet of cells held together by cell–cell junctional proteins. The adherens junctions, comprising the E-cadherin/β-catenin complex, are essential for survival. The tight junctions, which form the apical-most complex regulate ion and nutrient flow by acting like a molecular sieve. Thus, the epithelial barrier relies on tight junctions and adherens junctions to regulate permeability and maintain tissue integrity. Upon exposure to oxidative stress via peroxide, superoxide, hydroxyl, nitric oxide, or other ROS, the tight junctions and adherens junctions are first disrupted and rearranged [12,43,48,49,50,51,52,53,54]. In vitro studies using Caco-2 cells and immunofluorescence microscopy reveal ROS-induced reorganization of tight junction complexes, leading to decreased transepithelial resistance and paracellular permeability. Similarly, AJs undergo structural alterations, weakening epithelial integrity. Oxidative stress also disrupts the F-actin cytoskeleton, impairing the localization and function of ZO-1, Occludin, and the E-cadherin/β-catenin complex [49,51,54,55,56,57,58,59]. These disruptions facilitate pro-inflammatory signaling and contribute to inflammatory bowel disease and sepsis. While mechanistically, stress-related post-translational modification (PTM) in some tight junction proteins like Occludin has been elucidated, much needs to be deciphered with the other tight junction and adherens junction proteins to understand the stress-incurred damage to cell monolayers.

## 4. Oxidative Stress and Signal Transduction

Reactive oxygen species, including hydroxyl radicals, peroxides, and superoxides, regulate cellular signaling. However, in excess, these free radicals disrupt epithelial integrity and activate stress-response kinases. Hydrogen peroxide activates c-Src, leading to F-actin polymerization, cytoskeletal contraction, and junctional disruption, increasing the permeability of epithelial and endothelial cell monolayers, as well as also inducing neuronal degeneration [50,54,57,58,60,61,62]. ERK 1/2, another MAP kinase, is also required for peroxide-induced barrier dysfunction. Among stress-activated kinases, JNKs or SAPKs are activated by ROS through MAP kinase kinases (MAPKKs). Depending on the cellular context, JNK signaling can drive either apoptosis or survival [63,64,65].

This review delves into the specific roles of JNKs in the oxidative stress-activated disruption of the epithelial barrier function, especially in the epithelial cells of the gastrointestinal tract.

## 5. C-Jun N-Terminal Kinases (JNKs)

The c-Jun N-terminal Kinases have been identified as a part of a cascading series of discoveries of different MAPKs, such as ERK 1 and 2. However, JNKs are differentiated based on their more potent response to induced cellular stress in cycloheximide-treated rats, leading to their classification as SAPKs [66]. As derived from the name, JNKs phosphorylate and activate c-Jun, a component of the activator protein-1 (AP-1) complex [67], which regulates cell growth, apoptosis, and tumorigenesis, as well as intersects with all major signal kinases and transducers, like Src, Akt1, EGFR, and STAT3 [68,69,70,71]. Notably, the JNK pathway was found to be independent of Ras, Raf, and MAP kinase kinase (MAPKK), as demonstrated in IL-1-stimulated HepG2 hepatoma cells and growth factor-treated gingival fibroblasts [9].

JNK activation is mediated by MAPKKs, specifically MKK4 and MKK7, which phosphorylate the Thr-Pro-Tyr (T-P-Y) motif [72]. Upstream regulators of these MAPKKs include kinases activated by small GTPases of the Rho/Cdc42 family. Upon activation, JNKs translocate to the nucleus, where they phosphorylate important transcription factors, such as AP-1, Bcl-2, p53, and Elk-1. While JNKs are primarily activated by stress signals, ERK MAPKs generally promote cell survival and proliferation [73,74].

JNKs exist in three genetically distinct isoforms: JNK1, JNK2, and JNK3. Each gene is expressed as 3–4 different splice variants, together making 10. It is not known whether each splice variant has unique functions, which is a matter of investigation. JNK1 and JNK2 are ubiquitously expressed, whereas JNK3 is primarily found in the brain, testes, and pancreatic β-islet cells [75,76,77,78,79].

JNK1 was first discovered as a protein kinase stimulated by UV light and the oncoprotein Ha-Ras. It was shown to phosphorylate the c-Jun activation domain that activated AP-1 transcriptional complex [80,81]. Kryiakis et al. soon followed with a publication in *Nature* with their discovery of the stress-activated protein kinases (SAPKs) from cycloheximide-treated NIH3T3 cells and rats [66], which later became synonymous with JNKs. Dubbed p54, there were four variants that were detected in that study. Among the first reports for JNK-dependent stress-induced apoptosis, there came a study of the ceramide secretion in bovine endothelial and human monoblastic leukemia cells [82]. Ceramide release initiates apoptosis through the SAPK/JNK cascade. Different kinds of stress results in ceramide generation, like ionizing radiation, cytokine treatment (TNFα), heat shock, hydrogen peroxide, and UV-C treatment, all of which cause c-Jun phosphorylation [83].

JNK2 was discovered as a tyrosine-phosphorylated protein in mammalian cells stressed with lipopolysaccharide (LPS) [84]. Kallunki et al. cloned the first human JNK2 isoform [85], showing that it binds c-Jun 25 times more efficiently than JNK1 due to a unique β-strand region near the catalytic pocket (discussed in Section 6).

JNK3, specific to the brain and testis, was discovered as a “neuro specific MAP kinase”, p49(3F12) [86,87]. This neuronal-specific isoform of JNK has been associated with various neuronal functions and pathologies, including the hypoxic and ischemic damage in hippocampal neurons.

The focus of the remainder of this review is to examine the link between the stress and epithelial junctions where JNK signaling plays a vital role at the crossroads. We will also cover the structural features of JNKs and their implications in discovering JNK-targeted inhibitors for controlling stress-related tissue injury/damage. We will then discuss the role of JNK1 and JNK2–, including their activation under distinct kinds of stress and the consequences thereof, as well as the challenges in targeting this pathway with rationally designed inhibitors.

## 6. Structural Features of JNKs

**Figure 2:**
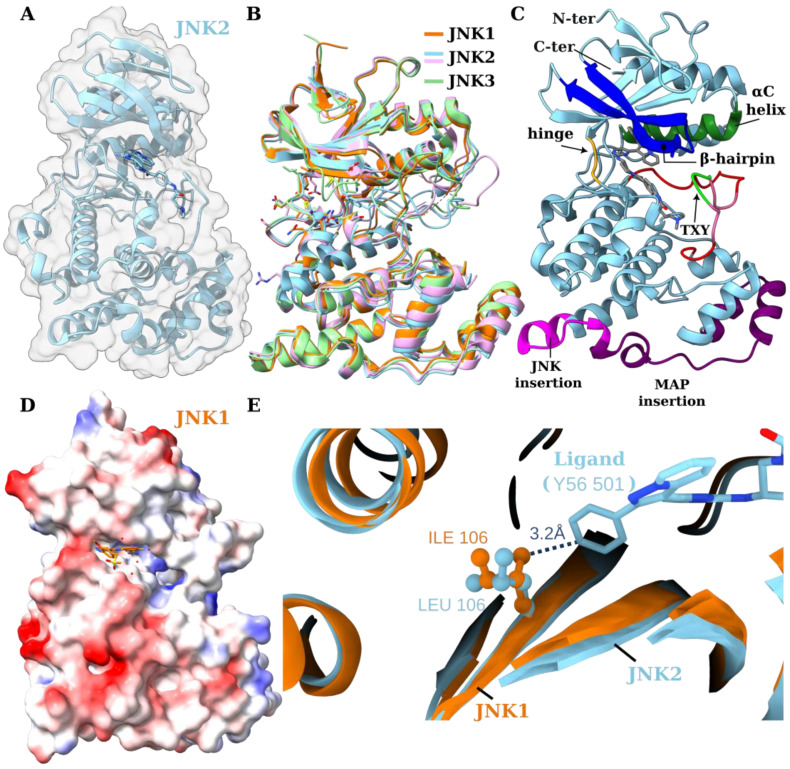
The overall structure of JNK1–3 kinases. In this figure, JNK1 is depicted in orange (PDB ID 3ELJ); JNK2 is depicted in cyan (PDB ID 8ELC) or pink (PDB ID 7N8T); and JNK3 is in green (PDB ID 4W4V). (**A**) A ghost surface representation showing the secondary structure of JNK2. (**B**) The structures of all three JNK1–3 aligned, showing the flexibility of the activation loop and β-hairpin. (**C**) Structural features of the JNK and MAP family color coded and then mapped onto JNK2. The hinge region, β-hairpin, and the αC helix are colored orange, blue, and dark green, respectively. The L16 containing the activation loop and the T-X-Y motif are depicted in red and pink and green, respectively. The MAP insertion is depicted in purple, and it contains a terminal JNK insertion (depicted in magenta). (**D**) A Coulombic (electrostatic) isosurface representation of the JNK1 showing the hydrophobic active site. (**E**) The I106/L106 residue in the ball and stick representation in JNK1 and JNK2, respectively, in proximity of the JNK2 selective ligand.

**The overall structure of JNK kinases and the characteristics of the ATP active site.** Being members of the MAPK family, the c-Jun N-terminal kinases (JNK 1-3) not only have a common overall structure that is similar to other kinases in the family, but they also possess the general structural features found in kinases. As with other kinases, the JNK1–3 is composed of two domains: an N-terminal domain and a C-terminal domain (Figure 2A–C). The two domains are connected by a hinge region, with the ATP site located at the deep cleft present between the two domains and in proximity to the hinge region. The N-terminal domain is predominantly composed of beta sheets, while the C-terminal domain is mainly composed of alpha helices. The structures of the three JNK kinases are very similar (Figure 2B).

Figure 2C illustrates the different structural features of the JNK kinases. The ATP analogs co-crystallized with JNK structures [88,89,90] were partially buried in the predominantly hydrophobic binding site (Figure 2D), and this site was bound by a glycine-rich β-sheet–loop–β-sheet hairpin (β1L0 and β2L0), which is hereafter referred to as the β-hairpin, the hinge region, the αC helix, and the activation loop. A portion of the ATP is accessible to the solvent. The flexible activation loop (also termed phosphorylation loop/lip, residues Ser217-Thr226) could reach out to the β-hairpin and the neighboring helix (αC helix), effectively closing the active site and trapping the ligand (Figure 2C). This loop held a T-X-Y motif that underwent either dual phosphorylation by MKK4 and MKK7 to activate the JNK enzymes or dephosphorylation by Serine/Threonine and Tyrosine phosphatases for deactivation [91]. The loop plays a critical role in the phosphorylation of the activated proteins. The flexibility of the activation loop is key for the substrate phosphorylation to occur and plays a critical role in binding to inhibitors at the ATP site. The glycine-rich loop of the β-hairpin was also found to be flexible and interacted with the ligands at the ATP site.

**Strategies for the design of JNK inhibitors.** JNK kinase inhibitors have mainly been used to target the ATP site (ATP-competitive inhibitors) or other sites (ATP non-competitive inhibitors). The former inhibits the activity of JNK by competing with ATP and, hence, prevents the phosphorylation of JNK substrates. Unfortunately, the high similarity between the ATP sites of kinases poses a challenge and, hence, complicates the design of specific inhibitors to these enzymes [92]. The limited specificity of such inhibitors often results in undesired effects or toxicity. A known example of this class of inhibitors is anthrapyrazolone [91], which showed effectiveness in Parkinson’s in mouse models, but it was also found to inhibit thirteen other kinases, reflecting poor specificity toward the JNK proteins [91].

The second class of inhibitors, which are ATP non-competitive, bind to other sites on the enzyme and do not interfere with ATP binding. Such sites are generally aimed at inhibiting protein–protein interactions. This may include the JNK substrate protein being phosphorylated (JNK-mediated phosphorylation) or other proteins upstream or downstream the signal cascade [92,93,94]. The inhibition of JNK-mediated phosphorylation has been demonstrated in the literature using minimal peptides that mimic the JNK binding domains (JBD). An example of such inhibitors is pep-JIP1 (an 11-amino-acid peptide), which illustrates an effective and selective reduction in the JNK activity [95] found in JNK substrates, such as the JNK-interacting protein-1 (JIP1). The interference with the latter protein–protein interactions inhibited the downstream signal in the MAPK pathway [96]. Like other members of the MAPK, other sites have been proposed as targets for inhibitors. These include the DFG-out site, the FXFP binding site, the CD-docking groove, and the backside site [97].

**Specific features of MAP kinases and the subgroup JNK kinases compared to other kinases.** Several features are characteristic of MAP kinases. In particular, MAP kinases possess several structural features that are not found in other kinases, including the β-hairpin, the MAP insert region (which involves an extended region after helix G, consisting of the helices α1L14 and α2L14) and αL16 (a C-terminal helix). It has been shown that the MAP kinase insertion is required for its interaction with other regulatory proteins. It has also been demonstrated that the activation loop exhibits multiple conformations, with Y185 and T183 being solvent-exposed or buried. Such dynamics may affect the activation of JNK kinases.

JNK3 has a 45% and a 51% sequence identity with ERK2 and p38 (within the homologous region), respectively [90]. It is, therefore, not unexpected that JNK kinases share common structural features with other MAP kinases. The architecture of MAP kinases, such as ERK2 and p38, is generally similar to JNK kinases, though with several key differences. Some of the main differences are that the JNK has extension and insertion regions (Figure 2C). Further, there is a slight geometric difference among MAP kinases, where the relative rotation of the two domains (i.e., N-and C-terminal domains) show 2.5° and 4° deviation from the JNK3 in ERK2 and p38, respectively, upon the superposition of the C-terminals of JNK3, ERK2, and p38 [91]. Moreover, the T-X-Y motif is present in all MAP kinases where X is a proline residue in JNK, glutamate in ERC2, and glycine in p38. It has been proposed that upon the phosphorylation of T221 and Y223, the two residues may interact with (Arg107 & Arg188) and (Arg227 & Arg230), respectively [90]. Moreover, the terminal 12 residues (Leu317-Lys328) in the MAP insert region (residues 283–328, purple and magenta-colored regions in Figure 2C), which are known as the JNK insert [90], are present in JNK kinases and absent in other MAP kinases. The JNK insertion extends the αH helix and forms an extra 310 helix.

## 7. The Role of JNKs in the Intestinal Epithelium

The gastrointestinal (GI) tract has a continuous sheet of epithelial cells that form the inner lining that faces the food ingested from the mouth, and it is tasked with digestion and absorption. The intestinal epithelium that begins with the duodenum is responsible for most, all the way through to the colon that absorbs water and electrolytes, of the absorption [98]. The GI mucosal barrier system is multilayered: First, mucin and other polysaccharides form a lining that limits contact of the epithelium to luminal microbes [99]. Second, the epithelium itself is tightly held as a sheet due to cell adhesion proteins, the apical most of which are the TJs, which function as a semi-permeable seal. In typical homeostatic conditions, the TJs regulate the entry of specific macromolecules and electrolytes but not toxins. TJs comprise tetra-spanning membrane proteins, namely claudins and Occludin, which are connected to the actin cytoskeletal network by the cytoplasmic side scaffolding protein zonula occludens-1 (ZO-1) [100]. Disruption of the tight junctions results in the withdrawal and recycling of claudins and Occludin [61,101]. This tight junction disruption creates a pathway for larger molecules to cross the cell–cell junctions, including potential toxins (especially endotoxins like LPS) [12,102]. This leads to a systemic immune response and increases the susceptibility of other organs to inflammatory damage (like the liver and brain) and to the risk of sepsis [22].

There is abundant evidence for the tight junction disruptive activity/signaling for JNKs. In keratinocytes of the oral mucosal epithelium, induction of JNK phosphorylation by Anisomycin disrupts ZO-1 staining within 2 h of JNK activation. Inhibiting JNK signaling with SP600125 (pan-JNK inhibitor) resulted in the restoration of ZO-1 and claudins at tight junctions [103]. Extracellular calcium depletion, which causes cell–cell junctions to pull apart, was accomplished through the activation of JNK signaling [104]. Other studies also indicate direct regulation of tight junctions by JNK activation. IL-6 induces JNK phosphorylation and the activation of the AP-1 complex, which transcriptionally activate claudin-2 expression (i.e., a claudin that allows paracellular flux of cations), increasing permeability [61]. Across the blood–brain barrier, TNFα-mediated JNK activation decreased the claudin-5, Occludin, and ZO-1 levels to increase barrier damage [105].

**JNKs and their interaction with the cytoskeleton:** JNKs can phosphorylate a variety of cytoskeletal protein substrates, but they can also be regulated by F-actin and Actin Binding Proteins (ABPs). An extensive review by Benoit et al. [106] covered the JNK substrates of F-actin, ABPs, intermediate filaments, and Septins. Conversely, JNK activation can also be triggered by the mechanical tension induced by Cdc42-dependent actin remodeling. F-actin depolymerization can also be an activator of JNK signaling. In keratinocytes of the oral mucosa, ROCK inhibition abrogated JNK phosphorylation and tight junction disruption, as well as stopped F-actin remodeling through the ERM linker proteins [103].

JNK1 and JNK2, though ubiquitously expressed in all cell types, have different effects on the epithelium. JNK1-KO, but not JNK2 or JNK3, knockout mice have a deficiency in skin epithelial barrier wound repair. In a study by Koehler et al., JNK1-KO wounds healed slower than WT, but JNK2 or JNK3 KO did not affect the barrier closure [107]. The authors suggested that JNK1 may be involved in epithelial differentiation, but it could also be as a consequence of increased cell proliferation and migration, leading to faster closure of wounds in the presence of JNK1. In myoblast cells, JNK1, but not JNK2, mediated TNFα-induced cell proliferation by inhibiting myoblast cell differentiation and promoting the generation of inflammatory cytokines such as IL-6 and LIF [108]. JNK1 activation is also known to promote cell survival through the induction of autophagy, as shown in a study by Wei et al. [109]. Under conditions of cell starvation, JNK1, but not JNK2, could phosphorylate Bcl-2, leading to disruption of the Bcl-2/beclin-1 complex and activating an autophagy response.

In many cell types, while the activation of a c-Jun-mediated AP-1 transcriptional complex is an important aspect of stress-induced JNK activation that drives cell fate toward apoptosis [69,70,110,111], the intestinal epithelium seems to be more peculiar. Stappenbeck et al. [59] showed that Rac1, using mice expressing constitutively active Rac1, led to the accumulation of p-JNK in the apical cytoplasm, and not in the nucleus, of proliferating and differentiating intestinal epithelial cells. This was associated with F-actin reorganization. In this case, c-Jun phosphorylation was not increased in the nucleus either. Adult crypts were elongated and showed increased proliferation, and these were accompanied by an abnormal distortion in the villus architecture. Rac1 or pJNK activation, however, did not increase any cell apoptosis rates, nor did it cause dysplasia, suggesting that the role of pJNK is not to cause apoptosis. In this study, however, the phosphorylated JNK detection was not specifically determined for any JNK isoform due to the lack of antibodies specific to pJNK1 or pJNK2. This continues to be a problem in the detection and activation of the JNK1 and JNK2 isoforms till date; as such, most studies that determine the activity of a specific JNK isoform utilize knockdown or knockout.

Several years later, Sancho et al. [111] developed transgenic mice that expressed a constitutively active JNK1 protein as a “JNKK2-JNK1” fusion protein [112]. In these mice, though c-Jun phosphorylation was increased in crypt base stem cells (CBC stem cells), JNK signaling, rather, increased intestinal cell proliferation and villus length. C-Jun was found at the cells at the tip of the villus. JNK1 was also found to increase levels of transcription factor 4 (Tcf4), a major component of Wnt-signaling pathway. JNK1 overexpression also accelerated tumorigenesis in an azoxymethane–dextran sodium sulfate (AOM-DSS) colitis mouse model of colon carcinoma. Overall, the above two studies indicate a pro-cell proliferation role for JNK signaling, particularly JNK1. It is of note that other studies with JNK1 deletion (JNK1−/−) showed a spontaneous development of tumors in the small intestine, but not in the colon [113]. This shows tissue-specific functions for JNKs that can only be investigated by developing tissue-specific transgenic mice.

JNK2, on the other hand, responded by activating upon various stressors in or at the intestinal epithelium. SAPK2 (p38 kinase) is involved in the H_2_O_2_-mediated oxidate stress activation of focal adhesions in endothelial cells, but it is distinct from JNK2 [47,114]. SAPK2 or p38 was found to be activated by MAPKAP (MAP kinase-associated protein kinase)-2 [47], whereas JNKs are activated predominantly by MEK4 and MEK7.

**Figure 3:**
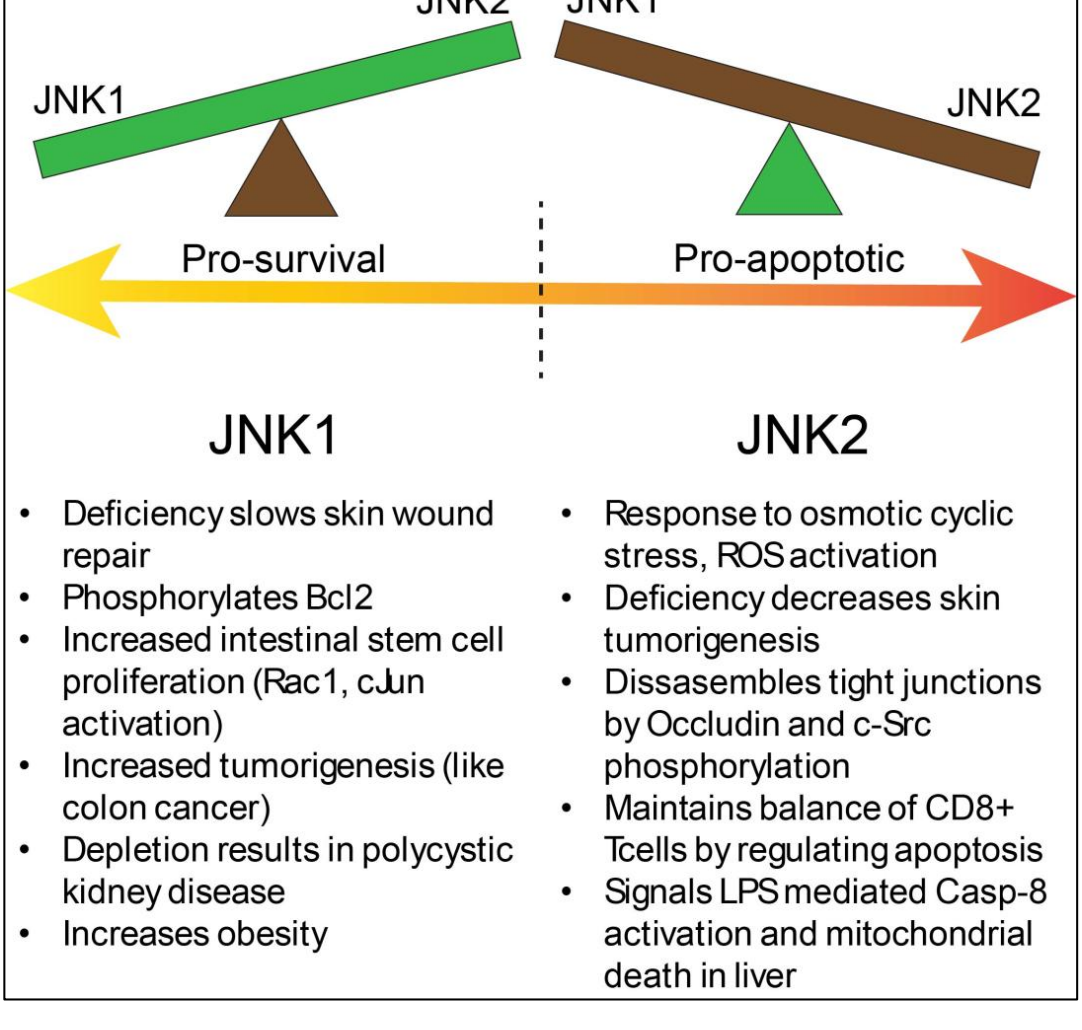
Summary of the differences between JNK1 and JNK2 functions. The organ-specific differences between JNK1 and JNK2 show that, in response to stress, JNK1 activates pro-proliferative signals, often skewing toward abnormal proliferation or cancer, whereas JNK2 activates pro-apoptotic signals both in and outside of the nucleus.

Samak et al. first showed that, in Caco-2 cells, inducing apical osmotic stress activates JNK2 specifically [58]. The lack of involvement of JNK1 has been verified by small interfering RNA (siRNA)-mediated knockdown studies. Anti-sense RNA to JNK2 inhibits tight junction disruption, similar to JNK inhibitor treatment, but JNK1 inhibition does not rescue tight junctions. In a follow up paper, the same authors discovered that intracellular calcium release is downstream of osmotic stress, which leads to JNK2-dependent tight junction disruption [29]. Depletion of extracellular Ca^2+^, i.e., thapsigargin-mediated ER Ca^2+^ depletion, or a knockdown of the calcium-transport Cav1.3 channels, all lead to downregulation of JNK2 phosphorylation. Using JNK-specific inhibitor SP600125 or calcium sequestration by BAPTA reverted tight junction disruption. That study was also the first to show phosphorylation of the tight junction protein Occludin in response to osmotic stress, which could be blocked by attenuating JNK and/or c-Src phosphorylation, suggesting crosstalk between stress-activated-JNK and c-Src kinase signaling pathways. This was also applicable to cyclic stress-activated Caco2 cells, as well as to ROS production by treatment with dextran sodium sulfate (DSS), suggesting that the JNK2 response is similar to the chemical, osmotic, or mechanical stress that leads to ROS production [56,57]. In the studies mentioned above, it is worth noting that pJNK signal (which is not specific to JNK1 or JNK2, it is instead inclusive of both) was found to be activated almost exclusively at or near the tight junctions but not in the nucleus. It is possible that c-Jun activity might not be involved here, with other proteins instead being targeted (Occludin in this case). Figure 3 provides a summary of the reciprocal nature of the relationship between JNK1 and JNK2, which has been discussed above.

## 8. Role of JNK1 and JNK2 in IBD

In a seemingly contraindicative study of an ulcerative colitis-like DSS model of colitis, JNK1-KO mice fared like WT mice when subject to repetitive DSS insult [115]. JNK2-KO mice, however, fared worse than their WT controls, with higher disease activity scores and increased mortality. This is contrary to the overall well known anti-inflammatory actions of drugs that inhibit JNK1-JNK2 activation. The study concluded that this could be due to the prolonged activation of cytotoxic T-cells in JNK2-KO that were otherwise destined for apoptosis since JNK2 is required for efficient T-cell activation and apoptosis but not for normal lymphocyte development. This could also be due to the potential tissue damage that occurs consequentially due to the hyperproliferation of CD8+T-cells in JNK2-KO mice [116], which has been shown to occur because of the increased IL-2 cytokine expression in these mice. Thus, in IBD, JNK1 does not significantly affect the intestinal epithelium, while JNK2 suppression leads to immune cell proliferation by dysregulation of apoptosis, leading to inflammatory activation. The pro-apoptotic action of JNK2 in the intestinal context seems to keep the immune cells at check.

Phospho-JNK, as well as p38 kinase, levels are elevated in the colonic epithelium in both Crohn’s disease [117] and ulcerative colitis [118,119]. In an ulcerative colitis-like DSS chemical colitis model, treatment with SP600125, a pan JNK inhibitor with affinity in the nanomolar range, and an inhibition of JNK resulted in a significant reduction in histopathological scores. This was associated with a specific decrease in the colonic and mesenteric lymphocyte CD23/CD28-stimulated TNF-α levels [119]. The phospho-JNK levels in infiltrating immune cells (macrophages) were higher in untreated compared to SP600125-treated cells [120]. It also affected the epithelial side, where epithelial apoptosis was reduced in the colon epithelia. It is not known yet if JNK1- or JNK2-specific inhibition would work better than a pan-JNK inhibitor. For this, the intestinal epithelial cell-specific contribution of the JNK isoforms would need to be parsed out, as well as, secondly, the generation of a JNK1/JNK2/JNK3 specific inhibitor, which has been a major impediment until today.

## 9. Differential Role of JNK1 and 2 in Epithelial Cells of Other Organs

**Skin (wound healing):** In the skin epithelium, JNK2 deficiency inhibits skin tumorigenesis, whereas, in JNK1-KO mice, it results in a higher tumor burden. Indeed, overall gene expression profiles of mouse fibroblasts lacking either JNK1 or JNK2 are markedly different from each other and their WT controls. JNK2-KO increases the expression of tumor suppressors, while JNK1-KO increases the expression of anti-apoptotic genes [121,122,123].

**Liver disease:** Wang et al. [124] showed that a tumor necrosis factor-induced toxic liver injury (LPS model) occurred because of the JNK2-, but not JNK1-, dependent activation of caspase-8 and the mitochondrial death pathway, i.e., JNK2-KO mice were protected from LPS-mediated liver injury. This was unrelated to c-Jun kinase activity, which was intact in the JNK2-KO mice but abolished in the JNK1-KOs. JNK signaling is implicated to be in both stages of NAFLD (non-alcoholic fatty liver disease). Mice lacking JNK1 and JNK2 in the liver were protected from Steatosis in diet-induced models of obesity, but this did make them susceptible to chemically induced hepatocarcinoma.

**Renal disease:** The JNK signaling pathway also plays a critical role in renal diseases and renal fibrosis. JNK activation has been shown in kidney parietal epithelial cells. In a well-designed approach to study the effect of JNK signaling on autosomal dominant polycystic kidney disease (ADPKD), Smith et al. first showed polycistin-2 (Pkd2) deletion activates JNK2 signaling, as well as c-Jun phosphorylation levels [125]. JNK inhibition, and particularly JNK1 and not JNK2 kidney-specific deletion, is necessary for reducing the severity of the cystic phenotype in juvenile mutant mice, which is achieved by inhibiting tubule epithelial cell proliferation. This again highlights a comparatively pro-proliferative role for the JNK1 isoform, unlike JNK2. In an ischemia/reperfusion model of injury (IRI), JNK activation occurs quickly, and treatment with inhibitors like SP600125 offers protection against tubular damage, but not when given a 1 h post insult [126].

**Obesity and diabetes:** The JNK pathway has been investigated in obesity and diabetes models and how it can contribute to insulin resistance. Activated JNK can directly phosphorylate insulin receptor substrates (IRS) 1 and IRS2 [8,38,79]. JNK1-KO mice have been found to be resistant to diet-induced obesity and have shown increased insulin sensitivity, which correlates with the reduced IRS1 phosphorylation at Ser307, the target for JNK, in the liver. In the pancreatic β-cell, it has been shown that JNK-mediated cytokine induction leads to cell death. However, contrary to the ubiquitous isoforms of JNK1 and JNK2, JNK3 is predominantly expressed in these cells [127]. Silencing JNK3, however, leads to an opposite outcome—enhancing the sensitivity of the β-islet cells to cytokine-induced apoptosis. JNK3 might, thus, be playing a stress-activated, protective role in these cells [38,79].

## 10. Summary of JNK1 Versus JNK2

There is no clear consensus on the mechanism behind differential activities and functions that JNK1 and JNK2 are associated with. Sabapathy et al. showed that levels of c-Jun, its kinase activity, and its AP-1 activity was higher in JNK2-KO fibroblasts than JNK1-KO, and this was attributed the c-Jun kinase activity on the JNK1 control [65]. JNK2 destabilized c-Jun levels, and a JNK2-KO prevented this degradation, leading to increased proliferation rates, which is a crucial function for the c-Jun complex. However, two years later, JNK2 was shown to positively regulate c-Jun in a different study by Jaeschke et al., who used a “chemically inactivated” JNK2 functional inhibition model [122]. Overall, JNK1 and JNK2 may both have redundant biochemical activities, but the activation of each JNK in vivo is dependent on the type of injury/insult, cell-type, and the kind of damage that the cells have sustained due to the insult. In addition, both JNK1 and JNK2 phosphorylate targets apart from c-Jun; thus, they are not restricted to regulation of the transcriptional activity inside the nucleus [78,94,126,128,129]. For example, JNK2 may be involved in phosphorylating the tight junction protein Occludin and reorganizing the actin cytoskeleton independently of its binding to c-Jun and AP-1 transcription factors [57,130,131,132]. This may very well be a biomarker for JNK2 activation, but it is not known whether JNK2-dependent Occludin phosphorylation occurs ubiquitously in all cell types or only in known disease states.

In summary, our take is that both JNK1 and JNK2 signaling can be contextual depending on the cell type or organ of interest that are particularly studied. Conditional knockouts and/or knock-ins might provide more insight into the tissue-specific roles of JNK1 and JNK2. In a whole organism knockout model, JNKs might play seemingly opposing roles in one cell type versus another. For example, in the case of JNK2-KOs, ROS stress response to DSS might be attenuated in the colonic epithelium, but normal upkeep of T-cells by apoptosis might also be reduced, causing more inflammatory responses. Conditional knockout specific studies could also be very useful in designing specific inhibitors and determining their route of administration. Finally, the development of isoform-specific JNK inhibitors becomes a major challenge since the signaling mechanisms are cross-connected, with JNK playing one role in one cell type but a different role in a connected cell-type: for example, the intestinal epithelium is protected from oxidative stress damage if JNK activity is silenced, but its localized immune cells proliferate without inhibition to damage the epithelium further by secreting pro-inflammatory cytokines.

## 11. Selective Inhibition of the Distinct Types of JNKs: Structural Cues

The above discussion warrants the use of JNK isoform-specific inhibitors, such as those that would selectively target JNK1 or JNK2. It is evident that the ubiquitous JNKs perform distinct roles in different circumstances, either pro-apoptotic or pro-proliferation. Since JNKs affect cell fate in multiple ways, it is not surprising that both JNK upregulation or downregulation affect a variety of cells, organs, and organ systems, contributing to disease pathophysiology. There are several positive and, at the same time, several challenging aspects of targeting JNK isoforms for treatment of human diseases. Despite the structural flexibility of these isoforms, it is still possible to develop JNK isoform-specific inhibitors.

Unfortunately, to date, no inhibitor developed thus far has singularly targeted a specific isoform. This is especially the case with respect to JNK1 and JNK2 targeting inhibitors. A review by Wu et al. [133] summarized the available inhibitors for inhibiting JNK signaling and their affinity to JNK1, JNK2, and JNK3, along with their current or potential use for specific diseases, which are under due process, either as clinical or pre-clinical trials. The inhibitor CC-90001, in clinical trials for pulmonary fibrosis, is perhaps the closest to being JNK1-specific, but it only has an 8-fold selectivity over JNK2 [134,135,136,137,138]. SP600125 was one of the earliest JNK signaling inhibitors produced, and it inhibits all JNK isoforms strongly [88,139]. This had raised interest in the design of selective inhibitors against specific JNK proteins. While a majority of the chemically synthesized inhibitors target the ATP binding site, there are some peptide mimics that prevent JNK binding to the JNK interacting protein (JIP) [74,95]. The latter approach of designing peptides shuts off the transcriptional activation of certain JIP-activated downstream promoter complexes like ATF-2, c-Jun, and Elk. Nonetheless, as detailed in previous sections, JNKs can target many more protein substrates outside of the nucleus without requiring interaction with JIP, thus preventing its use where JNKs act to directly phosphorylate functional proteins, like with the tight junction protein Occludin.

The main challenge, however, in the design of selective inhibitors is that the structure of the three JNK proteins are very similar. This is not surprising since the JNK1 and JNK3 PDB sequences (PDB IDs 1UKH [90] and 1JNK [88]) have 80% and 84% sequence identity, respectively, with that of JNK2 [89]. Notably, the ATP binding site differs among the three kinases in only one or two residues. Specifically, JNK1 and JNK2 differ in only two residues (98% identity, wherein Met77 is replaced by Leu77 and Ile106 by Leu106), whereas JNK2 and JNK3 differ by only a single residue (Leu77 to Met115) [140]. These minor discrepancies pose a challenge in designing selective inhibitors to the different JNK proteins. Thus, the key challenges in designing isoform-selective JNK inhibitors mainly lay in that the isoform are highly homologous and hence the lack of isoform-unique binding pockets that can selectively binding to specific isoforms. The fact that currently targeted sites have a high percentage of sequence identity among all the three JNKs impedes the rational design of such selective molecules. Moreover, the dynamic nature of the binding pockets greatly limits traditional computational docking methods and can result in unexpected outcomes.

Lu et al. designed and studied a series of inhibitors, several of which showed significant selectivity (>20-fold) for JNK2/3 over JNK1. In their study, they solved the structure of the JNK2 with AMP (a non-selective inhibitor) and YL2056, which is a covalent inhibitor that potently and selectively inhibits JNK2 over JNK1 (PDB IDs 7N8T and 8ELC, respectively) [140]. To further determine the basis of selectivity, they docked the inhibitor to other JNK-like proteins and carried out MD simulations of the three different JNK-YL2056 complexes. Based on these studies, the authors proposed several factors that may contribute to the selectivity of a compound to the three kinases.

First, Leu144 (Leu106 in JNK1), which is present in JNK2 and JNK3 (as shown in Figure 2E and was generated using ChimeraX [141]) and substituted in JNK1 (to Ile106), makes strong hydrophobic interactions with the ligand in the JNK2 crystal structure, but the ligand does not mimic that site in a JNK1-docked structure. This was attributed to the steric clashes by the Ile106 residue in the latter. Second, the Val54 backbone interaction in JNK2 with the ligand may have been perturbed by the substitution of this residue by Ile in JNK1,3. The authors proposed that such a bulkier side chain in JNK1,3 may have limited the motion of the main chain to enable the accommodation of the ligand in the binding pocket, as evident by the shift of the position of the β-sheet to accommodate the ligand in JNK2. More importantly, the authors noted that the activation loop and β-hairpin were highly flexible in their MD simulation and that Arg50 in JNK1 (which corresponds to Arg88 in JNK3 and is substituted by Ile in JNK2) interacts with Glu109 (corresponds to Glu147 in JNK3) [140], which may influence the dynamics of the β-hairpin. This hypothesis was confirmed by repeating the MD simulation with the JNK2 mutant (I50R). The latter showed that the β-hairpin moves away from the ligand, suggesting that this interaction (Arg50/88-Glu109/147) is likely to impact the ligand binding by affecting the dynamics of the β-hairpin.

**Figure 4:**
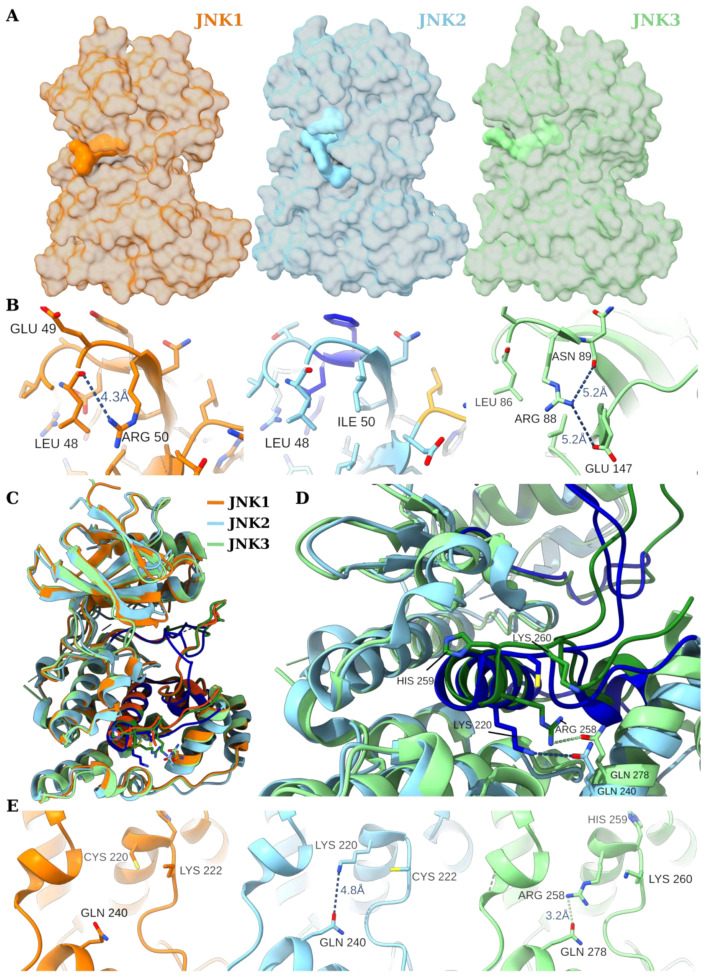
The proposed Interaction Sites 1 and 2 in JNK1 and JNK2. As in Figure 2, the JNK1 is depicted in orange (generated from PDB ID 3ELJ), JNK2 is depicted in cyan (generated from PDB ID 8ELC), and JNK3 is in green (generated from PDB ID 4W4V). (**A**) Isosurface representation of the protein with their corresponding ligands showing that the ligand was significantly buried under the surface. (**B**) A close-up view of Interaction Site 1. (**C**) A JNK1–3 aligned structure, showing the corresponding segments of the structure that were affected by the nature of Interaction Site 2, which are depicted in dark orange, blue, and green, respectively. (**D**) Inset of C with JNK1 hidden for clarity, showing the shift in the structure caused by the substitution of residue 258. (**E**) A close-up view of Interaction Site 2 (see text for details). The figure was generated using UCSF ChimeraX [141].

Further, structural flexibility also determines JNK inhibitor selectivity. Taking a closer look at the bound ligands to all three kinases in various crystal structures (Figure 4A), it is clear that the ligand is substantially buried under the surface of the protein. More specifically, the ligand is trapped by two dynamic parts (or two arms) of the protein; the activation loop and the β-hairpin. Thus, for the ligand to enter the pocket and take the observed crystal structure pose, the two dynamic arms must move away from the binding pocket, allowing the protein to adopt an open conformation and then move to trap the ligand to form a closed conformation. It is evident from the sequence alignment of the three proteins and from the discussion above that the interactions of residues R/I/R 88 (in JNK 1/2/3, respectively) with Glu147 or with the backbone of residue 86 affect the motion of the β-hairpin (designated here as Interaction Site 1) (Figure 4B).

Further, the interaction of the (named Interaction Site 2 here) residue CHK/KGC/RHK (residues 258–260) seen in JNK 1/2/3, respectively, with the residue Q278, was found to play a similar role in controlling the motion of the segment of residues (206–266), including the αL12 helix, i.e., the L12 loop that contains the activation loop and αF helix (Figure 4C,D). The strength and the level of interaction at Site 2 clearly control the extent by which L12 can move. The latter, in turn, controls the motion of the L12 (or the activation loop) and its ability to reach out to trap the ligand. Figure 4E (generated using ChimeraX [141]) illustrates these interactions in detail. K258 strongly interacted with Q278 in JNK2, making it more compact and essentially pulling the interacting helices downward, whereas, in JNK3, R258 and Q278 also strongly interacted, but only after enforcing a slightly different conformation compared with JNK2, which occurred due to the longer length of the Arginine residue. On the contrary, this interaction was completely lost due to the substitution of the positively charged R258/K258 in JNK2/3 with C258 in JNK1. Further, K260 was not found to interact with Q278 in JNK1 as the shortest distance between the two residues was >8 Å, making the residues (206–266) more labile to movement in JNK1 compared to its counter parts in JNK2/3. This may explain the observed distinct selectivity of JNK2/3 from JNK1 for ligands targeting the ATP site [140].

In summary, the two Interaction Sites 1 and 2, remotely and indirectly, controlled the ligand stability at the active site by either enabling or limiting one or both of the protein dynamic arms (β-hairpin and activation loop structures) to surround the ligand in the active site, hence affecting the ligand stability by their (direct) interaction. The various combined effects of the two interactions can give each of the three enzymes a unique and distinct capacity to specifically select certain ligands. The effect discussed here is an example of how minor sequence variations (a few residues) that are distant from the active site can induce a dynamic signal that enable the enzymes to bind selectively to distinct substrates and inhibitors. These variations may well offer a route to design unique inhibitors that target one specific JNK isoform, which could offer a lot of promise for diseases in which JNK signaling can be targeted. To this end, understanding the structural flexibility of JNKs is likely to play a pivotal role in the design of novel inhibitors in the future. Such a design could benefit from discovering the alternative binding pockets or allosteric pockets that are district among JNK isoforms. Moreover, this design could enormously benefit from recent advances in computational drug design. Methods like Induced Fit Docking [142], Free Energy Perturbation [143], MD-base ensemble docking [144,145], and machine learning [146] could be used to consider the unique dynamic signature of the different JNK isoforms in the drug design process.

## 12. Conclusions

In conclusion, the broad functional spectrum of JNK activity, its diverse phosphorylation targets, and its ubiquitous expression make JNK1 and JNK2 essential stress-activated kinases with context-dependent effects on the cell fate. Depending on the nature of the injury/stress and cellular context, JNK activation can lead to either pro-survival or pro-apoptotic outcomes, contributing to the pathophysiology of various diseases. Therefore, the development of isoform-specific JNK inhibitors holds great promise for advancing targeted therapies with enhanced efficacy and reduced side effects.

## Data Availability

There was no new data generated in this manuscript.
